# Analysis of peritumoral hyperintensity on pre-operative T2-weighted MR images in glioblastoma: Additive prognostic value of Minkowski functionals

**DOI:** 10.1371/journal.pone.0217785

**Published:** 2019-05-31

**Authors:** Yangsean Choi, Kook Jin Ahn, Yoonho Nam, Jinhee Jang, Na-Young Shin, Hyun Seok Choi, So-Lyung Jung, Bum-soo Kim

**Affiliations:** Department of Radiology, Seoul St. Mary’s Hospital, College of Medicine, The Catholic University of Korea, Seoul, South Korea; South-Central University for Nationalities, CHINA

## Abstract

**Objectives:**

The extent of peritumoral tumor cell infiltrations in glioblastoma contributes to poor prognosis. We aimed to assess additive prognostic value of Minkowski functionals in analyzing heterogeneity of peritumoral hyperintensity on T2WI in glioblastoma patients.

**Methods:**

Clinical data (age, sex, extent of surgical resection), O6-methylguanine-DNA methyltransferase (MGMT) promoter methylation status and pre-operative T2WI of 113 pathologically confirmed glioblastoma patients (from our institution, n = 61; from the Cancer Imaging Archive, n = 52) were retrospectively reviewed. The patients were randomly grouped into a training set (n = 80) and a test set (n = 33). Peritumoral T2 hyperintensity was manually segmented and Minkowski functionals—a texture analysis method capturing heterogeneity of MR images—were computed as a function of 11 grayscale thresholds. The Cox proportional hazards models were fitted with clinical variables, Minkowski functionals features as well as both combined. The risk prediction performances of the Minkowski functionals and combined models were validated on a separate test dataset. The sex-specific survival difference of the entire cohort was analyzed according to MGMT methylation status via Kaplan-Meier survival curves.

**Results:**

Thirty-three Minkowski features (11 area, 11 perimeter and 11 genus) for each patient were acquired giving a total of 3729 features. Cox regression models fitted with clinical data, Minkowski features, and both combined had incremental concordance indices of 0.577 (*P* = 0.02), 0.706 (*P* = 0.02) and 0.714 (*P* = 0.01) respectively. The prediction error rate of the combined model—having clinical and Minkowski features—was lower than that of Minkowski functionals model (0.135 and 0.161, respectively) when validated on a test dataset. No sex-specific survival difference was found according to MGMT methylation status (male, *P* = 0.2; female, *P* = 0.22).

**Conclusions:**

Minkowski functionals features computed from peritumoral hyperintensity can capture heterogeneity of glioblastoma on T2WI and have additive prognostic value in predicting survival, demonstrating their potential in complementing currently available prognostic parameters.

## Introduction

Glioblastoma is the most common primary malignant brain tumor [1] notorious for its aggressive clinical course and poor prognosis. Even with the current standard therapy of surgical resection followed by concurrent chemoradiation with temozolomide, the median survival after initial diagnosis of glioblastoma is around 18–24 months [2]. Making appropriate prognostic evaluation on initial diagnosis is therefore important for risk stratification in glioblastoma patients.

Prior studies demonstrated that clinical factors including age and extent of tumor resection [3] as well as O6-methylguanine-DNA methyltransferase (MGMT) promoter methylation status were prognostic factors associated with glioblastoma [4, 5]. Recent advances in radiomic feature-based imaging analysis paved the way for identification of various imaging features as prognostic markers [6–9], which may soon allow non-invasive prognostic evaluation prior to surgical resection.

Extensive peritumoral T2 hyperintensity on conventional MR images is known to be associated with poor survival outcome [10–13]. Peritumoral edema in glioblastoma contains infiltrative tumor cells and thus making it more susceptible to recurrence [14, 15]. At the molecular level, peritumoral edema heterogeneity is known to contribute to poor survival in glioblastoma [16]. Such heterogeneity can also be appreciated on MR images as it leads to hyper- and hypointensity in T2WI [17].

We postulated that Minkowski functionals (MF) as image descriptors would be able to capture heterogeneity within peritumoral T2 hyperintensity at levels not easily discernible to the radiologist. While sharing some commonalities, MF distinguishes itself from other texture analysis techniques in that it employs binary images with multiple levels of increasing thresholds to remove pixels rather than direct examinations of MR images [18]. In particular, the uniqueness of MF lies in its ability to characterize multidimensional objects into 2D space of area, perimeter, and genus which corresponds to shape, structure and connectivity, thereby allowing parameterization of image heterogeneity as well as analysis of underlying tissue biology reflected as morphological information [19].

This study presents an approach of texture-based image analysis using MF on T2WI. Our study aims to determine MF’s potential additive prognostic value when combined with commonly available clinical data in predicting overall survival (OS) of treatment-naïve glioblastoma patients.

## Materials and methods

The institutional review board of The Catholic University of Korea, Seoul St. Mary’s Hospital approved this retrospective study (approval number: KC18DESI0497) and the requirement for informed consent was waived.

### Study population

Between December 2008 and November 2017, a total of 203 patients with pathologically confirmed primary glioblastoma from our hospital were retrospectively reviewed. The inclusion criteria were as follows: 1) pre-operative MR images with available axial T2WI and gadolinium(Gd)-enhanced T1WI; 2) availability of individual OS information. The exclusion criteria were: 1) previous surgery or biopsy (n = 9); 2) loss of follow-up or unknown survival status (n = 16); 3) no MGMT promoter methylation status (n = 32); 4) no concurrent chemoradiation therapy (n = 67); 5) no peritumoral T2 hyperintensity (n = 9); and 6) follow-up period shorter than 12 months in living patients (n = 9). A total of 61 patients meeting the criteria were included.

The external cohort consisted of a total of 53 pre-operative multi-parametric MR scans from the TCIA, which is an open archive of various oncologic medical images and clinical database created by the National Cancer Institute. The inclusion criteria for this cohort were: 1) availability of pre-operative MRI sequences (Gd-T1WI, T2WI); 2) presence of peritumoral T2 hyperintensity; 3) availability of individual overall survival information and 4) availability of individual MGMT status. Overall, a total of 113 cases were included in the study.

After combining both cohorts, all patients were randomly dichotomized into a training and test set (7:3 ratio with *n* = 80 in the training set and *n* = 33 patients in the test set). The distribution of survival status was kept balanced between both sets. OS was defined as duration from the initial MR imaging-based diagnosis to death or the last follow-up in alive patients. The extent of surgical resection was grouped into gross total removal—radiologically defined as no visible enhancing lesion within 48 hours after surgery or disappearance of all peritumoral T2 hyperintensity seen on preoperative MRI—and non-total removal for all non-gross total removal. The summary of patients’ characteristics is listed in Table 1.

**Table 1:** Demographics and clinical characteristics of patients.

Characteristic	Training Set (n = 80)	Test Set (n = 33)
Gender		
Male	44(55%)	22(67%)
Female	36(45%)	11(33%)
Age, median (range)	59 (25–78)	57 (31–78)
Extent of Resection		
Total	41(51%)	17(52%)
Non-Total	39(49%)	16(48%)
MGMT status		
Methylation	40(50%)	22(67%)
Unmethylation	40(50%)	11(33%)
Overall survival, median (range)	433 (54–2213)	448 (138–2125)
No. TCIA^a^	36(45%)	16(48%)
No. deaths	67	27

All patients, n = 113

^a^The number of patients from the Cancer Imaging Archive

### MRI acquisition and protocol

MR images were acquired on two different 3-Tesla (T) MR scanners (Magnetom Verio; Siemens, Healthcare Sector, Erlangen, Germany) with a 12-channel phased-array coil and Ingenia (Philips Healthcare, Best, the Netherlands) with a 32-channel phased-array coil. The acquisition parameters for both units were: 1) T2WI (repetition time (TR), 3300 msec; echo time (TE), 93 msec; flip angle (FA), 150; field of view (FOV), 210x210 mm; acquired matrix, 448x358; number of excitations (NEX), 1; echo train length (ETL), 17; section thickness, 5mm), 2) FLAIR (TR, 9000 msec; TE, 95 msec; FA, 90; FOV, 210x210 mm; acquired matrix, 384x307; NEX, 1; ETL, 17; section thickness, 5mm) and 3) Gd-enhanced T1WI (TR, 250 msec; TE, 3.0 msec; FA, 70; FOV, 210x210 mm; acquired matrix, 448x358; NEX, 2; ETL, 1; section thickness, 5mm). Postcontrast images were acquired immediately following gadobutrol injection (Gadovist; Bayer Schering, Berlin, Germany).

For the TCIA validation cohort, all MR images were acquired with a 1.5 T or 3.0 T scanners. The acquisition parameters were: 1) T2WI (TR, 3000–5500 msec; TE, 80–105 msec; FA, 90; FOV, 200x200 to 240x240 mm; acquired matrix, 256x192 to 256x224; NEX, 1–2; ETL, 8; section thickness, 3-5mm) 2) FLAIR (TR 10000 msec; TE 125–155 msec; FA, 90; FOV, 220x220 to 240x240 mm; acquired matrix, 256x192 to 320x224; NEX, 0.5–1; ETL, 28–32; section thickness, 2.5-5mm) and 3) Gd-enhanced T1WI (TR, 583–817 msec; TE, 8–12 msec; FA, 90; FOV, 200x200; acquired matrix, 256x192; NEX, 2; ETL, 2; section thickness, 4-5mm).

### ROI placement and image processing via Minkowski functionals

On T2WIs, ROIs encompassing entire peritumoral T2 hyperintensity were placed semi-automatically on each slice by blinded and blinded (5 years and 20 years of experience in neuroradiology, respectively) using NordicICE (version 4.1.3., NordicNeuroLab AS, Bergen, Norway). The contrast-enhancing portions of the tumor on Gd-T1WI were subtracted to selectively include non-enhancing T2 hyperintensity. In addition, any non-enhancing necrotic portions of tumors were selectively removed from ROIs. After reviewing ROIs, any discrepancies were settled by consensus of two radiologists.

**Fig 1:**
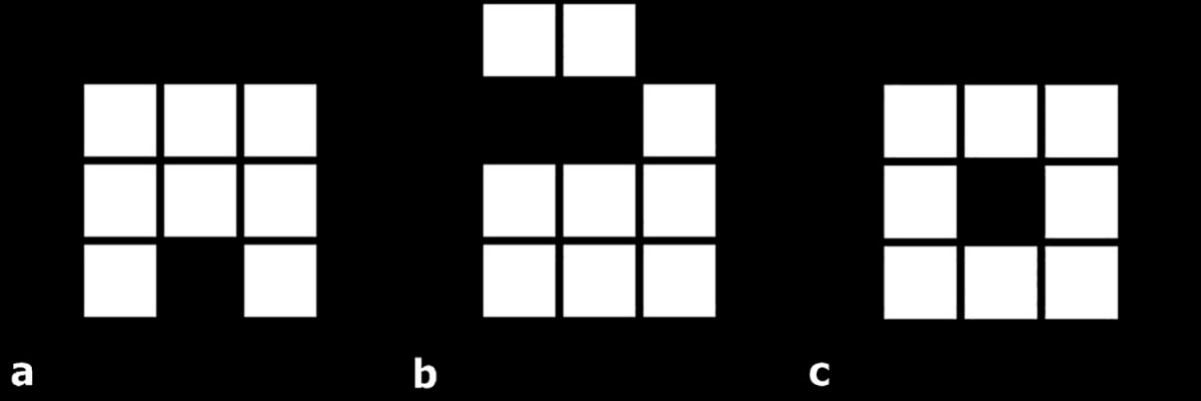
Representative images of 2D Minkowski functionals with area, perimeter and genus denoted as *A*, *U*, and *X*. For (a), the number of pixels, noncommunicating pixels, and noncommunicating vertices is 8, 23 and 16, respectively, giving *A* = 8, *U* = 14 and *X* = 1. The same calculation gives (b) *A* = 9, *U* = 18, and *X* = 1 and (c) *A* = 8, *U* = 16, and *X* = 0.

**Fig 2:**
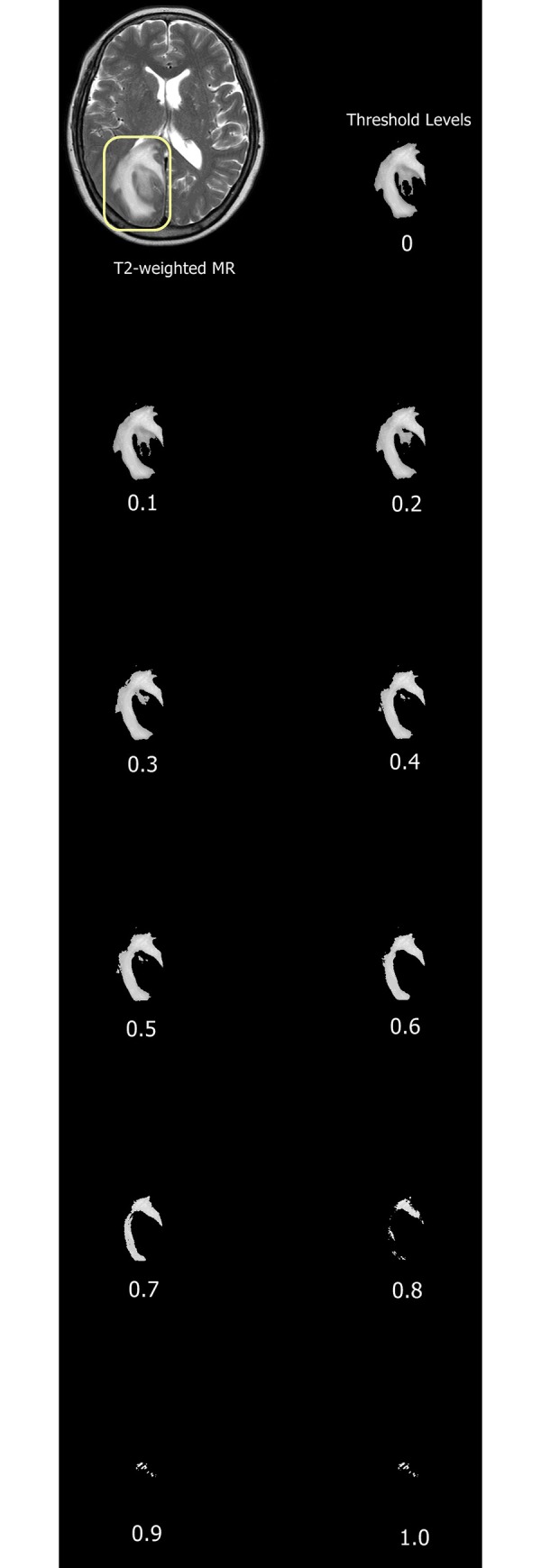
Representative images of peritumoral hyperintensity segmentation on axial T2WI. Binary images at 11 incremental threshold levels where each number represents threshold level with 0 set as the entire ROI while 1.0 is near blank.

From these segmented images, signal intensities were normalized to the surrounding brain parenchymal intensity after which MF features were computed using MATLAB (2018a, MathWorks, Natick, MA) image analysis code described previously [20], yielding binary images consisting of pixels with an intensity greater than a threshold ranging from 0 to 1. The first image is set as the whole ROI since it contains all pixels above 0 while the final image at 10^th^ threshold is set as empty. For each binary image, three different MF values of Area (*A*), Perimeter (*U*), and Genus (*X*) were calculated using the following equations [21]:

1. Area: *A* = number of visible pixels
2. Perimeter: *U* = −4Area + 2noncommunicating pixels
3. Genus: *χ* = Area − noncommunicating pixels + noncommunicating vertices,

where noncommunicating pixels and vertices are pixels without contiguous pixels [19]. A simplified illustration of *A*, *U*, and *X* is shown in Fig 1. Representative images of MF analysis are shown in Fig 2.

### Statistical analysis

Statistical analyses were performed with R statistical packages (version3.4.4, R Foundation, Vienna, Austria; www.R-project.org). Statistical significance was set at P < .05. Univariate Cox proportional hazards analyses on clinical data including age, sex, MGMT methylation status and surgical resection type were performed from which the significant variables were selected and used to build a multivariate model. We implemented backward elimination to choose meaningful MF features that were used in multivariate Cox regression analysis. After elimination of less significant variables, the model with the lowest Akaike Information Criterion was selected [22]. We computed concordance indices (C-index) for each of our three Cox regression models via Survival package in R. The C-index is a measure of how subjects are correctly ordered with respect to predicted survival times where C-index of 1 is a model with perfect predictive accuracy and C-index of 0.5 is a poor model having predictive accuracy equal to random chance.

To assess the performance of risk prediction, we tested the Cox regression models on the test set using prediction error curves over time and the integrated Brier scores (via ‘pec’ function from the pec library [23, 24]). The integrated Brier scores measure the accuracy of probabilistic predictions where a score of 0 is a perfect model and 0.25 is a non-informative model with a 50% incidence of the outcome. Finally, sex-specific survival analysis according to MGMT methylation status was performed via Kaplan-Meier survival curves with log-rank tests.

## Results

### Cox proportional hazards analysis on clinical data and Minkowski features

At univariate Cox proportional hazards analysis, age (hazard ratio = 1.02; 95% CI = 1.001–1.046; *P* = 0.04) and type of surgical resection (hazard ratio = 1.76; 95% CI = 1.071–2.899; *P* = 0.02) were found to be significant variables associated with survival while other two variables of sex and MGMT methylation status were not found to be significant (95% CI = 0.577–1.530; *P* = 0.8 and 95% CI = 0.4245–1.134; *P* = 0.15, respectively) (Table 2). Age and type of surgical resection as well as MGMT methylation status were fitted on a multivariate Cox regression analysis and named as the clinical model.

**Table 2:** Summary of Cox proportional hazards model of clinical variables.

	Univariate			Multivariate		
	HR	95% CI	*P* value	HR	95% CI	*P* value
Age	1.02	1.001–1.046	0.04	1.02	0.996–1.042	0.11
MGMT methylation status	0.69	0.4245–1.134	0.15	0.75	0.453–1.235	0.26
Surgical resection	1.76	1.071–2.899	0.02	1.67	1.009–2.756	0.04
Sex	0.94	0.577–1.530	0.8			

HR = hazard ratio; CI = confidence interval

**Fig 3:**
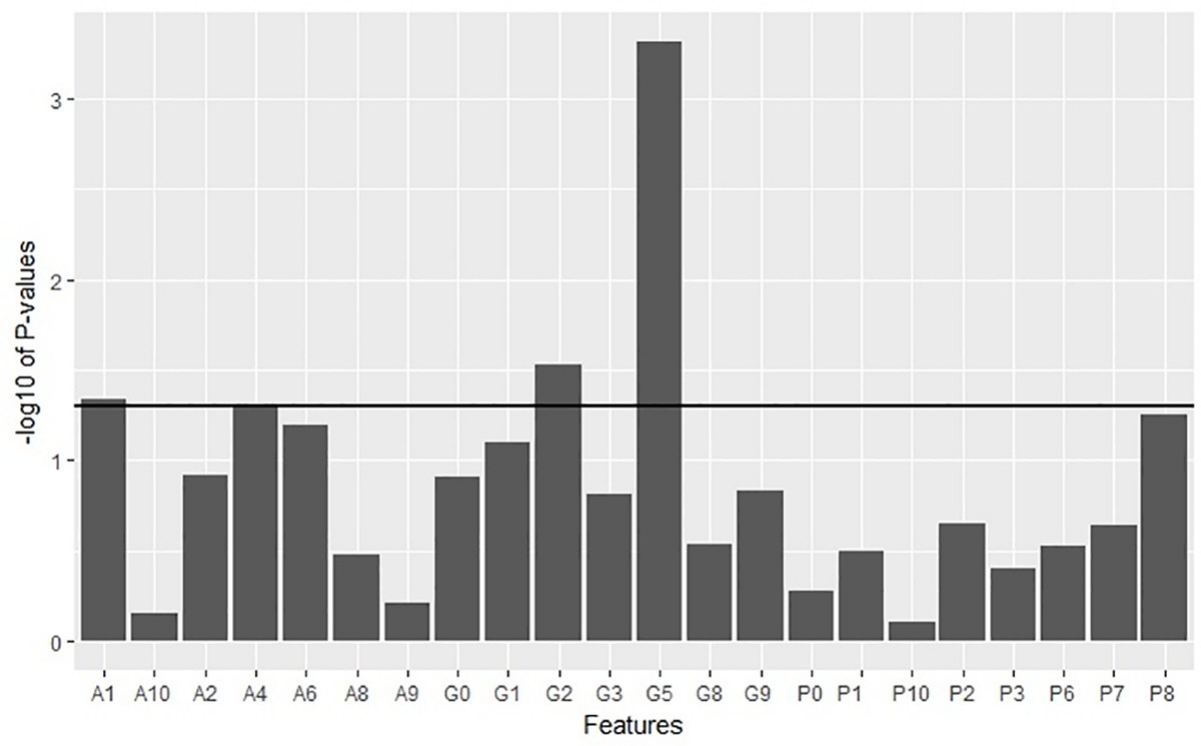
Graph of -log10*P*values for all features derived from the Cox regression model. The significance threshold is set at *P*<0.05 (horizontal line) (A, P, and G stand for area, perimeter and genus, respectively with corresponding numbers indicating levels of threshold).

As for the MF model, all MF features were initially fitted on a multivariate Cox regression model from which backward stepwise elimination was applied to reduce the number of features. A final model was derived with the lowest Akaike Information Criterion containing 22 MF features (7 Areas, 8 Perimeters, 7 Genus). Area at threshold level 1 (*P* = 0.0464), genus at threshold levels 2 (*P* = 0.029) and 5 (*P*<0.001) were found to be significantly associated with survival within the multivariate model. All the selected features were plotted with respect to *P* values derived from the Cox model (Fig 3).

### Survival analysis performances of clinical, Minkowski and combined models

The clinical and MF models yielded C-indices of 0.577 (likelihood ratio test, *P* = 0.02) and 0.706 (likelihood ratio test, *P* = 0.02), respectively. Combination of MF features and clinical variables (age, surgical resection and MGMT methylation status) was fitted on a multivariate Cox regression model that resulted in C-index of 0.714 (likelihood ratio test, *P* = 0.01). (Table 3).

**Table 3:** Concordance indices of clinical, Minkowski and combined models.

	C-Index	*P* value^a^
Clinical model^b^	0.577	0.02
MF model^c^	0.706	0.02
Clinical + MF model	0.714	0.01

^a^*P* values calculated from likelihood-ratio tests

^b^Clinical model: Cox regression model fitted with age, surgical resection and MGMT methylation status

^c^Minkowski functionals model

### Validation of MF model and MF + clinical model on test set via integrated brier score

**Fig 4:**
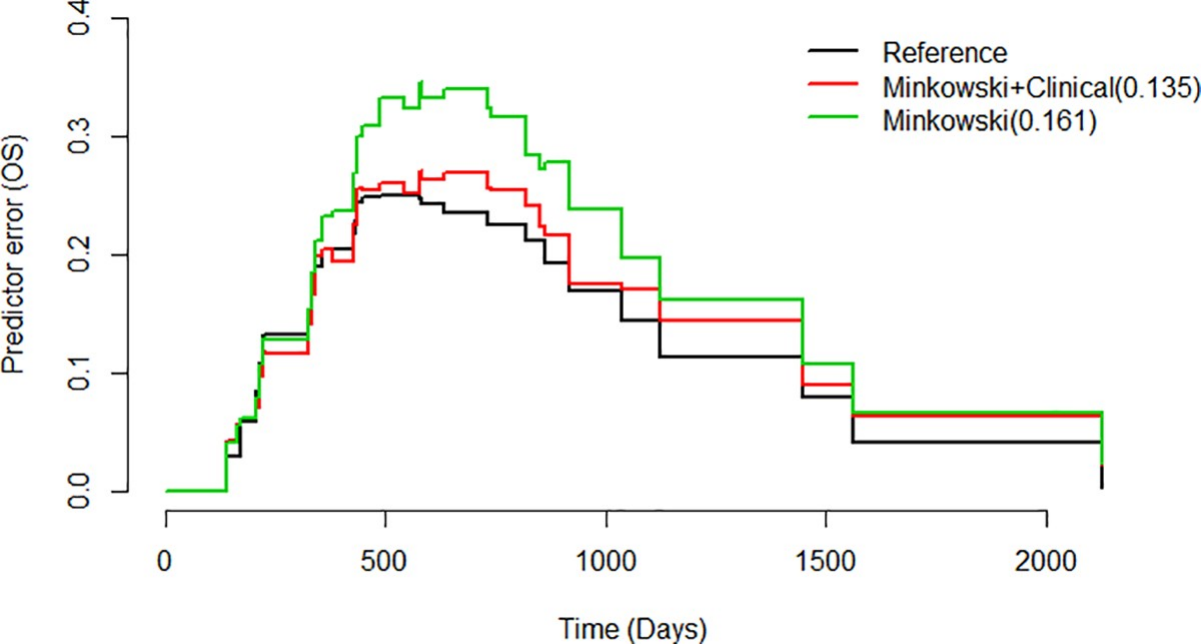
Prediction error curves of MF model, MF combined with clinical model and reference over survival time in days.

The prediction error curve of MF model resulted in an integrated Brier score of 0.161. When clinical variables were added onto optimal MF features, the prediction error curve resulted in lower integrated Brier score of 0.135 (Fig 4), indicating survival predictive performance closer to the reference model which had a score of 0.119.

## Sex-specific survival analysis based on MGMT methylation status

**Fig 5:**
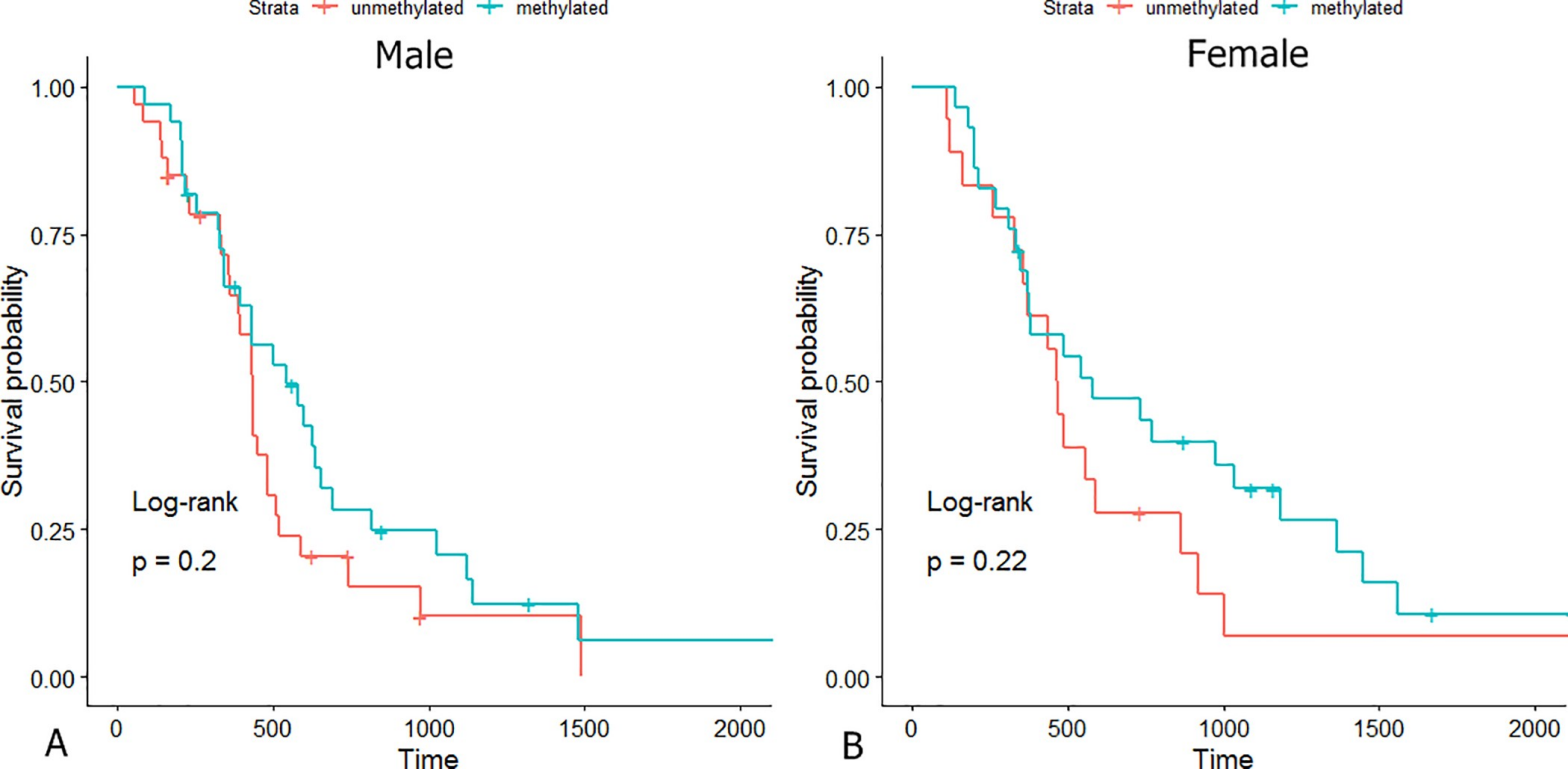
Kaplan-Meier survival curves according to MGMT methylation status of (A) male and (B) female cohort.

Survival analysis of the entire cohort demonstrated no sex-specific survival differences according to MGMT methylation status (Fig 5A and 5B).

## Discussion

In this study, we analyzed whether MF can capture heterogeneity of extensive peritumoral T2 hyperintensity associated with glioblastoma and its potential value as prognostic marker. Compared to a prior study on analyzing peritumoral infiltration of glioblastoma via MF histogram analysis [25], our study extended the scope by applying MF as texture analysis with emphasis on patients’ prognosis. MF were chosen as modality of texture analysis because of their relatively automated and reliable image analysis with parameterization of image heterogeneity [19]. We only applied 11 thresholded images per slice because previous studies showed that increased numbers of thresholds of over 11 provided no additional benefits [17–19]. Moreover, our dataset consists of two separate cohorts randomly combined, thereby increasing not only the sample size but also the possible heterogeneity of population investigated.

The results of this study are in line with a prior study by Liu et al. who demonstrated that local and regional heterogeneity of glioblastoma could be associated with survival rate [26], except that our study applied MF to specifically analyze peritumoral T2 hyperintensity heterogeneity.

The Cox proportional hazard model fitted with clinical variables including age, type of surgical resection and MGMT methylation status showed relatively low C-index, a survival predictive metric (Table 2). While MGMT methylation status was not found to be a significant factor in predicting survival, we nevertheless fitted it into the clinical model since it has been found to be an important prognostic marker of glioblastoma in a wide body of literature [5, 27, 28]. Interestingly, a few previous studies with a small sample sizes of glioblastoma patients also reported that MGMT methylation status was not significantly associated with survival [29–31]. Similarly, we believe that a relatively small number of glioblastoma patients in the training dataset might have attributed to such conflicting results. Of importance, emerging evidence suggest that there is a sex-specific survival difference according to MGMT methylation status [32, 33]. According to Schiffgens et al., male patients of three independent glioblastoma cohorts showed no survival difference based on MGMT methylation status while female patients showed survival difference [33]. However, our results did not find significant sex-specific survival differences, which may also be attributable to smaller sample size.

At univariate analysis, while the covariate age showed a minimally increased hazard ratio, the type of surgical resection was found to be a significant predictor of survival such that patients receiving non-total surgical resections had shorter overall survival (Table 2). Our finding is consistent with several studies that have shown the added survival benefit of gross total resection over other types of subtotal resections [3, 34, 35].

Peritumoral MF features combined with clinical variables resulted in improved prediction of glioblastoma survival as compared with prediction by MF features alone. The reduction of prediction error rate especially during survival days corresponding to one to two years may imply that the combined model is better in predicting long-term survivors (Fig 4). This additive value of MF in prognosis prediction is consistent with other similar recent studies on texture analysis [6, 9] except that their analyses were based on radiomics which is a field of quantitatively measuring radiographic imaging by computing complex texture patterns [36].

It is interesting to note that two genus features were found to be statistically significant from the Cox regression model at threshold level 2 and 5 (Fig 3). As previously reported by Fox et al., genus value suggests a greater variance and heterogeneous appearances in MR images of breast cancers [18]. A similar prior study on applying MF in differentiating pseudoprogression from progression in recurrent glioblastoma found that true progression displayed more heterogeneous MF features as non-uniform genus values than those of pseudoprogression [20]. Such findings are somewhat in line with the results of our study with regards to the genus features.

Our study introduced MF, a relatively new approach of analyzing image heterogeneity and applied it into prognostic models of predicting survival in glioblastoma patients. We strengthened the prognostic models via (a) incorporating an external dataset of TCIA of similar cohort size and (b) validating our models on a separate test dataset, thereby minimizing the possibility of overfitting often associated with data-driven predictive models.

There are several limitations in this study. On top of the inherent bias due to the retrospective design, only routine MR protocols (T2WI and Gd-enhanced T1WI) were used for this study; more multi-parametric MR protocols could have improved the survival predictive strength. In addition, variability of protocols and MR scanners, especially in the TCIA dataset—with their variable slice thicknesses, TR and TE—might have affected the consistency of the image heterogeneities. Finally, inclusion of more clinical variables such as Karnofsky performance status, or a well-known molecular prognostic marker like isocitrate dehydrogenase 1 (IDH1) mutation status could have further reinforced the clinical model in predicting survival outcomes. Particularly, survival prediction of glioblastoma based on combination of IDH1 and MGMT methylation status outperforms the prediction by either of the biomarkers [37, 38]. However, only a few patients in our cohort had IDH1 status available and subsequently it was not possible to include IDH1 for analysis. Future studies will need to investigate the potential roles of both biomarkers along with texture analysis.

## Conclusions

Application of MF as texture analysis may be useful in analyzing heterogeneity of peritumoral hyperintensity of glioblastoma on pre-operative T2WI. Furthermore, when combined with clinical variables MF features showed additive prognostic potential in predicting overall survival of treatment-naïve glioblastoma patients. We propose that MF carry a complementary prognostic role and should be investigated further in future researches.
